# A Calibration Method for Acoustic Space Charge Measurements Using Multilayer Samples

**DOI:** 10.3390/s18082508

**Published:** 2018-08-01

**Authors:** Guillermo Mier-Escurra, Armando Rodrigo-Mor, Peter Vaessen

**Affiliations:** 1 DC Systems, Energy Conversion & Storage, Department Electrical Sustainable Energy, Delft University of Technology, Mekelweg 4, 2628CD Delft, The Netherlands; A.RodrigoMor@tudelft.nl; 2 DNV-GL Energy, Utrechtseweg 310, 6812AR Arnhem, The Netherlands; Peter.Vaessen@dnvgl.com

**Keywords:** pulse-electroacoustic (PEA) measurements, dielectric measurements, charge measurements, space charge, calibration, multilayer dielectrics, dielectric interfaces

## Abstract

Nowadays, the use of several methods for the measurement of space charges in dielectrics has become more relevant. The availability of different space charge measurement methods brings the necessity of calibration and characterization of measurement equipment to reach standardized measurements. In this paper, a method is presented to apply known charges at built samples which can be used for acoustic and thermal methods to compare measured values with calibration purposes. Experimental tests for validation purposes were performed in flat samples by comparison of results between a single layer and multilayer samples. Moreover, this method can be used to measure the resolution and accuracy of space charge measurement systems.

## 1. Introduction

With the increasing use of high voltage direct current (HVDC) systems, the space charge phenomena are becoming more relevant. The space charge present in the insulation of electrical equipment, distort the electric field distribution and may lead to accelerated electrical aging and even breakdown [1,2,3,4,5,6].

The most widely used non-destructive methods for the measurement of space charges are the acoustic and thermal methods. These methods follow the principle of either exciting the charges by an electrical disturbance and measuring the mechanical response or the reverse method where the electrical response is measured when the charges are mechanically excited [3,7,8,9,10,11,12].

Due to the acoustic distortion and the transducer-amplifier response, the methods require mathematical post-processing to obtain accurate quantitative values [8,13,14,15,16]. The post-processing involves deconvolution methods whose calibration is obtained by measurement of known surface charge values at the electrodes when a known voltage is applied at a space charge free sample. Following this procedure, the existence of acoustic discontinuities for the generation and the propagation of the acoustic signals at the electrodes are not fully taken into account, which may affect the calibration [17,18,19].

In this paper, a method is presented for calibration purposes in which solid dielectric samples with known charge values, controlled by an external voltage source can be used for calibration and equipment characterization. In [10], corona charged samples were used for calibration and equipment characterization but concluded that the method is not suitable for calibration purposes because of inconsistency in the samples. In [20,21] a method is presented in which solid dielectric samples with known charge values controlled by an external voltage source, can be used for calibration and equipment characterization. A similar approach to this work is proposed and extended in this paper. The validity of using multilayer-samples to emulate known values of space charges in the dielectric is demonstrated in this document with the use of the pulse-electroacoustic (PEA) method. This paper is organized as follows: in Section 2, the theoretical background for the acoustic calibration, charge calculation, and their application is explained. In Section 3, the sample preparation and the test setup are described. In Section 4, the results of the tests and discussion are presented. In Section 5, the calculation of acoustic attenuation and dispersion factors using the presented method is elaborated, and in Section 6 its use for equipment characterization is proposed. Section 7 ends this paper with a conclusion.

## 2. Theoretical Background

As previously mentioned, the common calibration procedure involves the calculation of a transfer function using deconvolution processes. For the PEA method, deconvolutions are a common practice in the post-processing of the measurements. Using a space charge free sample with a known voltage, deconvolutions are performed using the actual measured signal and the expected calculated signal before electric and acoustic distortions. From this process, errors may arise, as the transfer function is calculated by comparing the pressure waves generated at the external electrodes, which have a different impedance mismatch than the signal coming from charges at the dielectric bulk. Equations (1)–(3) represent the pressure waves generated at the bottom electrode, top electrode, and the insulation bulk respectively, after traveling through the sample and transmitted to the bottom electrode:

(1) $${\tilde{p}}_{bot}{}^{\prime }\left(t\right)=G_{bot}{\tilde{p}}_{bot}\left(t\right)$$

(2) $${\tilde{p}}_{top}{}^{\prime }\left(t\right)=G_{top}T_{s-bot}k_{g}\left(d\right)F^{-1}\left[P\left(d,\omega \right)H\left(d,\omega \right)\right]$$

(3) $${\tilde{p}}_{s}{}^{\prime }\left(t\right)=G_{s}T_{s-bot}k_{g}\left(x\right)F^{-1}\left[P\left(x,\omega \right)H\left(x,\omega \right)\right]$$

(4) P(d,ω)=F[p˜top(t−dv)]

(5) P(x,ω)=F[p˜s(t−xv)]

(6) $$H\left(x,\omega \right)=e^{-\alpha \left(\omega \right)x}e^{-i\beta \left(\omega \right)x}$$

where ${\tilde{p}}_{bot}\left(t\right)$, ${\tilde{p}}_{top}\left(t\right)$ and ${\tilde{p}}_{s}\left(t\right)$ are the transient pressure waves [Pa] generated at the bottom electrode, top electrode and inside the sample respectively. F represents the Fourier transform, $k_{g}\left(x\right)$ is the geometric factor (in case of flat samples it can be considered equal to (1)). The factor α(ω) is the frequency dependent attenuation [neper/m] and takes into account the decrease of the wave magnitude while it travels through the medium. The factor β(ω) is the frequency dependent phase factor [1/m], which is the dispersion and takes into account that the speed of sound is frequency dependent [22]. The t, d and v represent time [s], the sample thickness [m] and the average value of propagation speed [m/s] of the acoustic wave across the sample for spatial location purposes. $G_{bot}$, $G_{top}$ and $G_{s}$ are the generation coefficients at the bottom and top electrode interface and at the insulation bulk respectively; as the generated pressure waves are divided and travel in two directions, but only the wave traveling towards the electrode with the transducer (bot electrode) is detected. $T_{s-bot}$ is the transmission coefficient at the bottom electrode due to the acoustic impedance mismatch. The generation and transmission coefficients can be calculated as [23,24]:

(7) $$G_{bot}=\frac{Z_{bot}}{Z_{s}+Z_{bot}}$$

(8) $$G_{top}=\frac{Z_{s}}{Z_{top}+Z_{s}}$$

(9) $$G_{s}=\frac{Z_{s}}{Z_{s}+Z_{s}}$$

(10) $$T_{s-bot}=\frac{2Z_{bot}}{Z_{s}+Z_{bot}}$$

$Z_{top}$, $Z_{s}$ and $Z_{bot}$ represents the acoustic impedances [kg·m^−2^·s^−1^] of the top electrode, the insulation bulk, and the bottom electrode respectively.

**Figure 1:**
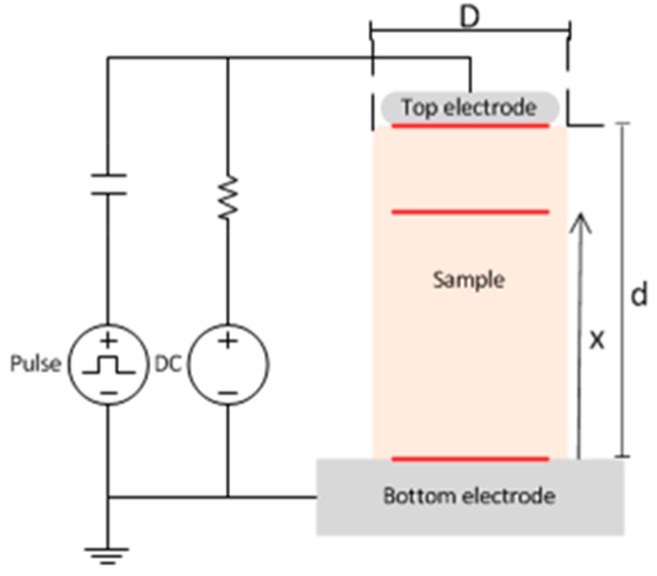
Schematic representation of a PEA measurement for a flat sample. The sample diameter is several times bigger than the height (D >> d) but the scale is modified for representation purposes.

As we can see in Equations (7)–(9), the fraction of the traveling pressure waves towards the sensor are different either if they are generated at the bot electrode, at the insulation bulk or the top electrode, ($G_{bot\;}\neq G_{top}\neq G_{s}$). This pressure waves are then affected by the transfer coefficient at the interface, compensating for the waves generated at the sample but not for the top electrode. Because the transfer function is commonly calculated using the external electrode signals, this might result in a deviation to the measured signals originated at the insulation bulk. The configuration of the electrodes are represented in Figure 1.

With the use of pressure waves generated at the insulation from known values of charges, transfer functions can be calculated which corresponds directly to the dielectric bulk. Other values such as acoustic attenuation factors might also be directly calculated by comparing known charge pressure waveforms at distinct locations of the sample, but it is not analyzed in this document.

### 2.1. Calculation of Charges

The method consists of a stack of dielectric layers with a nanometric thickness electrode at the dielectric-dielectric interfaces to form a single sample.

By having control of the geometry and material of the layers, the capacitances between the electrodes is known. The relation between capacitances, voltages, and charges at each electrode is represented in the following equation:

(11) $$\left(\begin{array}{c} Q_{1}\\ \vdots \\ Q_{N} \end{array}\right)=\;\left[\begin{array}{ccc} C_{11} & \cdots & C_{1N}\\ \vdots & \ddots & \vdots \\ C_{N1} & \cdots & C_{NN} \end{array}\right]\left(\begin{array}{c} U_{1}\\ \vdots \\ U_{N} \end{array}\right)$$

where the C matrix represent the mutual capacitances [F] between each electrode in the calibration sample, and the diagonal represents the capacitance of each electrode towards infinity. Q and U represent the charge [C] and voltage [V] respectively, at each of the electrodes of the calibration sample.

**Figure 2:**
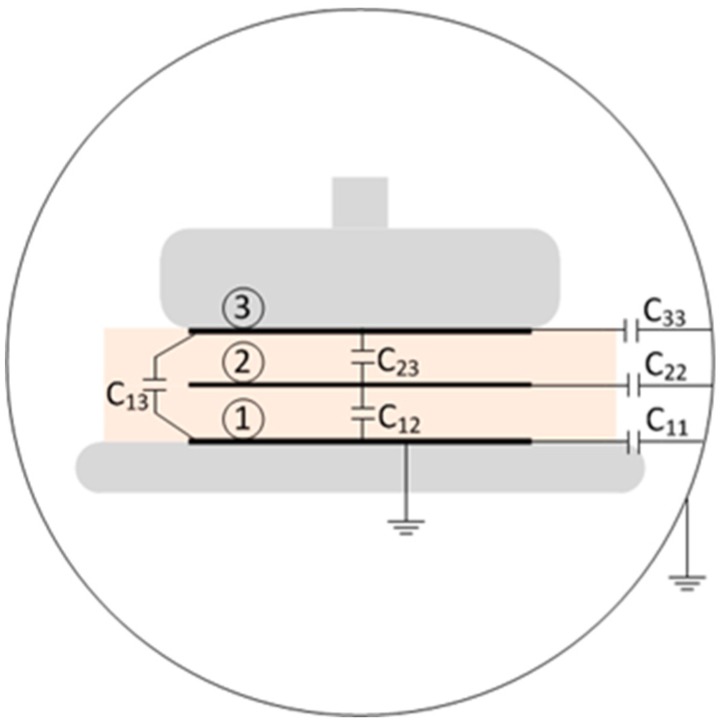
Two layers sample, with the representation of the bottom electrode (1), interface electrode (2), top electrode (3), and their mutual capacitances.

An example of a two layers sample and its electrodes are represented in Figure 2. In the figure, the outer circle represents a grounded spherical shell with an infinite radius. Even though the spherical shell at infinity and the lower electrode (represented as “electrode 1” in Figure 2) have the same grounded voltage, it is advantageous to consider it as an independent electrode, to calculate the existing charges in this electrode due to the voltage at the other electrodes.

### 2.2. Voltage Application at the Dielectric Bulk Electrode

As previously mentioned, the method consists in using a fixed voltage at the electrodes in the interfaces to generate known values of charges, and subsequently measure them as space charges using the acoustic methods for calibration purposes.

For the multilayer sample to resemble as much as possible a single layer dielectric with trapped charges, the interface should be as invisible for the mechanical wave as possible. This means that the interface electrode should have a thickness smaller than a tenth of the higher frequency wavelength component of the traveling acoustic signal used for the measurement. The small thickness allows us to neglect the acoustic interaction of the interface electrode and to avoid distortion in the acoustic signal due to differences in acoustic impedance and acoustic attenuation. A thin interface electrode also allows us to consider the different charges generated by the different mutual capacitances in the same electrode as an average value at the electrode position because of the limited resolution of the space charge measurement system in comparison with the thickness of the electrode.

In this paper, two methods to apply a voltage at the interface electrodes are proposed. First, a fixed electric connection between the interface electrode and a DC voltage source through a high resistance. Second, a temporal connection between the interface electrode and a DC voltage source. Each method has its strengths and weaknesses.

A fixed electric connection between the interface and the DC voltage source can modify the voltage value at any time and keeps the voltage constant during the whole measurement. The reason for the resistance is to avoid the free flow of charges during the transient of the space charge measurement. The RC time constant between the resistance and the sample capacitance should be several times higher than the pulse duration, so it does not affect the measurement. At the same time, it neglects the conductive path for the high voltage (HV) pulse in the case of the PEA method.

The temporal connection method consists of applying a voltage at the electrode and then physically disconnecting the voltage source without reducing the voltage, so the charges stay in the electrode. The advantage is that the high resistance is not necessary because the voltage source is not capable of providing charges during the transient, and there is not an alternative route for the voltage pulse in case of the PEA method. The disadvantage is the continuous depletion of charges at the interface because of leakage currents, which makes this method hard to apply.

## 3. Experimental Setup

For the experimental setup, measurement tests using the PEA method were performed. The fixed connection between the direct current (DC) voltage source and the interface electrode configuration was chosen. Two samples were used for comparison, a single layer epoxy as a reference and two epoxy layers in a sandwich arrangement with an electrode at the interface.

### 3.1. Sample Preparation

**Figure 3:**
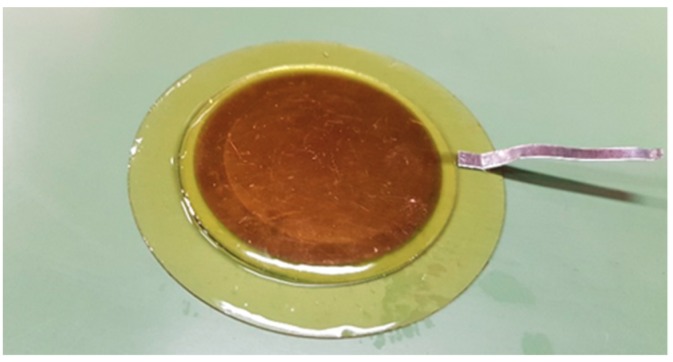
Dual layer epoxy sample with a 30 nm thickness gold electrode in-between layers.

The dual layer sample consists of two epoxy layers (Araldite MY 740, hardener HY 918, Huntsman), each one with a gold plating electrode of approximately 30 nm at the interface between layers, as can be observed in Figure 3.

The geometric characteristics of the samples are presented in Table 1 and Table 2. It can be observed that as the thickness has a variation of approximately 0.06 mm across the samples, this will result in a small measurement distortion which can be seen in the measurements of Section 4. A small amount of silicon oil was used at the interface to improve the acoustic contact.

### 3.2. Test Setup

**Figure 4:**
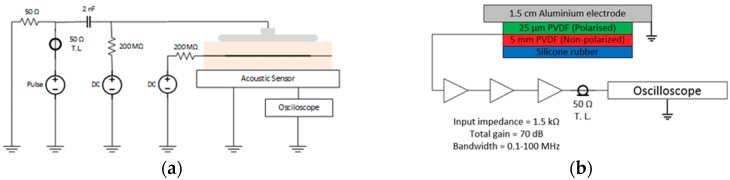
Test setup for the PEA method used in the multilayer sample. (**a**) Full test system. (**b**) The construction of the acoustic sensor.

The common arrangement for a PEA method was used with the difference of using a DC voltage source connected at the sample interface. An equivalent circuit of the setup is shown in Figure 4. The pulse is generated by the switching of a pulse generator switch (HTS 80-12-UF, Behlke). The used oscilloscope is a Waverunner 44 Xi-A 400 MHz (Lecroy). The acoustic sensor consists of a 25 µm film of polarized polyvinylidene fluoride (PVDF) backed by 5 mm of non-polarized PVDF which amplified voltage signal reaches the oscilloscope through a 50 Ω transmission line.

The expected charge-voltage relation at each of the electrodes was calculated with the aid of finite element software to get the values for the mutual capacitance matrix. The geometry in the model is the dual layer sample depicted in Table 1, the inner electrode was considered as infinitely thin; the relative permittivity used for the epoxy was 4.1.

**Figure 5:**
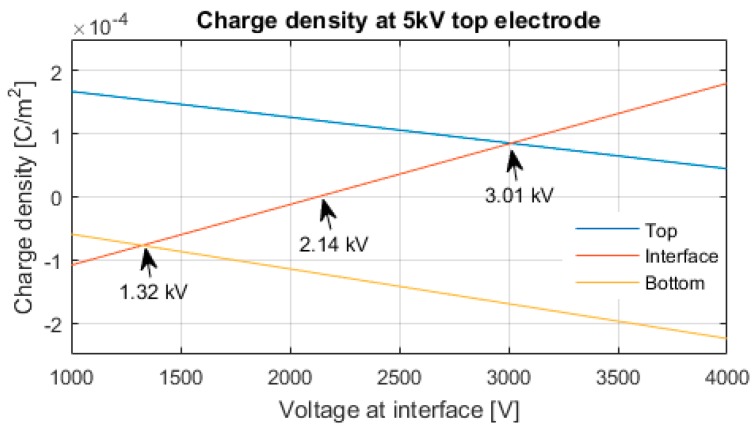
Dual layer sample interface voltage-charge relation for each electrode.

The charge values at different applied voltage levels are calculated using Equation (11). During tests, low voltage values were used to avoid space charge accumulation in the epoxy during the short duration of each test. Because of the interface electrode thickness, the charges at each electrode are treated as surface charge density instead of volume charge density, which is more common for PEA measurements. Figure 5 shows the relation of the surface charge density at each electrode and the voltage at the interface, keeping a fixed 5 kV voltage at the top electrode while grounding the bottom electrode.

In Figure 5, three interface voltage values that were used in the test are shown. With 1.32 kV at the interface electrode, the charge density at the bottom electrode is equal to the charge density at the interface. With 2.14 kV at the interface electrode, the charge density is zero. With 3.01 kV at the interface electrode, the charge density at the top electrode is equal to the interface charge density.

As seen in Table 1 and Table 2, the single layer electrode is thicker than the total dual layer sample. To make a meaningful comparison between the two samples, the electric field between the top and bottom electrodes should be the same to generate an equal amount of surface charges. To achieve this, the top electrode voltage and the pulse voltage used at the dual layer sample are multiplied by a $k_{d}$ factor for the single layer sample:

(12) $$k_{d}=\frac{d_{s}}{d_{d}}$$

where $d_{s}$ is the single layer sample thickness [m] and $d_{d}$ is the total dual layer sample thickness [m]. This means that the applied voltage at the single layer sample will be $k_{d}$ times the voltage at the external electrodes of the dual layer sample. The pulse voltage is also multiplied to this constant.

## 4. Experimental Results and Discussion

For all the tests, the duration of the measurements were less than 30 s. With the short duration of the tests and the low voltages used, no significant space charge is considered to develop. The measurements results are shown as the voltage signal without any post-processing involved, in order to compare the electric and mechanical distortions at each sample. Practically this means that the results are shown as the measured voltage signal at the oscilloscope and not in charge values.

For the experimental results, a comparison between the single layer sample and the dual layer sample at zero interface charge were performed. A voltage of 5 kV at the top electrode and 2.14 kV at the interface (see Figure 5) was used for the dual layer sample. For the single layer sample, following the Equation (12), 5.9 kV were used at the top electrode to keep the electric field equal (≈3.17 kV/mm) at both samples.

### 4.1. Comparison between Single Layer and Dual Layer Sample

**Figure 6:**
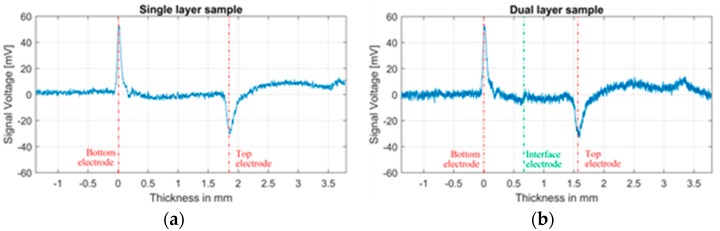
PEA signal without post-processing of 500 averaged measurements. (**a**) Single layer sample at 5.9 kV top electrode. (**b**) Dual layer sample at 2.14 kV interface and 5 kV top electrode.

From Figure 6a,b, we can compare the signal from the single and dual layers samples. It is observed that the signal voltage which represents the charges at the bottom electrode for both samples are equal. For the top electrode, the magnitude looks almost equal. Nevertheless, it can be observed that in the dual layer sample the value is slightly bigger. This difference is attributed to the smaller thickness of the sample which results in less traveling path for the acoustic signal and therefore has suffered less attenuation.

In Figure 6b, at the interface, small disturbance peaks can be distinguished which resembles heterocharges. This peaks can be attributed to two factors: First the non-uniform thickness of the built samples (not to be confused with surface roughness), which results in a not-completely uniform electric field across the interface electrode resulting in a measured signal even without the existence of space charges. The second factor is the polarization at the interface electrode, which even with the minimum thickness of the interface electrode in comparison to the resolution of the measuring system, it can be noticeable. The inhomogeneity of the interface such as the oil, and on a microscale the existence of oxidation layers, cavities and impurities between the layers can also produce the accumulation of charges [25,26,27]. Nevertheless, because of the short duration of the test and the low electric field, the accumulation is estimated to be negligible.

Even with the measured signal at the interface of the dual layer sample, the top and bottom electrode measured signals are consistent between both samples. Meaning that in the case of a non-zero total amount of charges at the interface of Figure 6b, the influence is not significant enough to affect surface charges at the electrodes.

It is worth to mention that the negative peak just after the bottom electrode peak, is not accumulated space charge, but the combination of the piezo-amp response and the direct response of the acoustic signal due to the non-ideal voltage pulse waveform which has a small undershoot. In the consecutive peaks, this pulse distortion cannot be seen because the acoustic losses have dissipated it.

### 4.2. Measurement of Generated Charges at the Interface Electrode

**Figure 7:**
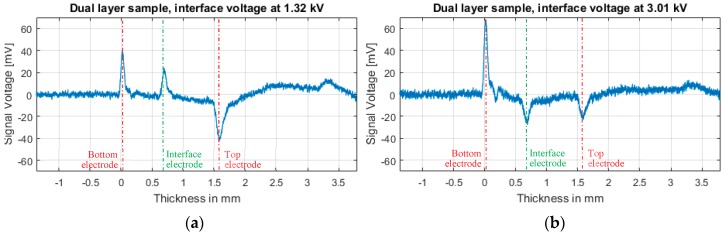
PEA signal without post-processing of 500 averaged signals, at 5 kV top electrode using the dual layer sample. (**a**) 1.32 kV at the interface electrode. (**b**) 3.01 kV at the interface electrode.

Figure 7a,b show the measured values in the dual layer sample with different voltages at the interface electrode. In Figure 7a we can observe the measured signal with the epoxy-epoxy interface voltage at 1.32 kV. With this voltage at the interface, the charge value at the epoxy-epoxy interface should be equal to the charge value at the bottom electrode. The measured difference in the experimental test is because of the acoustic attenuation of the material. The difference in the acoustic impedance mismatch at the bottom electrode-epoxy interface and the dielectric-dielectric interface should not affect the signal as it is shown in Equations (1)–(10) because both layers of the dielectric are of the same material. It can be explained in the following way:

(13) $${\tilde{p}}_{bot}\left(t\right)={\tilde{p}}_{s}\left(t\right)$$

Combining Equations (1) and (3) with (7)–(10), and neglecting the acoustic losses for a moment, it gives us:

(14) $${\tilde{p}}_{bot}{}^{\prime }\left(t\right)=\left(\frac{Z_{bot}}{Z_{s}+Z_{bot}}\right){\tilde{p}}_{bot}\left(t\right)$$

(15) p˜s′(t+xv)=(ZsZs+Zs)(2ZbotZs+Zbot)p˜s(t)

where x is the distance from the bottom electrode to the dielectric-dielectric interface [m]. As we can see, the coefficients are the same for both signals:

(16) $${\left(\frac{Z_{bot}}{Z_{s}+Z_{bot}}\right)}_{}=\left(\frac{Z_{s}}{Z_{s}+Z_{s}}\right)\left(\frac{2Z_{bot}}{Z_{s}+Z_{bot}}\right)$$

(17) ∴    p˜top(t)= p˜s′(t+xv)

At Figure 7b, where the inner interface is at 3.01 kV, the measured value at the epoxy-epoxy interface is not equal to the measured top electrode value. In this case, the acoustic attenuation and the acoustic impedance mismatch at the top epoxy-electrode interface and the dielectric-dielectric interface play a significant role, the measured signals are not equal, even though they have the same charge values. Following the same procedure as before and considering d as the distance between the top and the bottom electrode:

(18) $${\tilde{p}}_{top}\left(t\right)={\tilde{p}}_{s}\left(t\right)$$

(19) p˜top′(t+dv)=(ZsZtop+Zs)(2ZbotZs+Zbot)p˜top(t)

(20) $$\left(\frac{Z_{s}}{Z_{top}+Z_{s}}\right)\left(\frac{2Z_{bot}}{Z_{s}+Z_{bot}}\right)\neq \left(\frac{Z_{s}}{Z_{s}+Z_{s}}\right)\left(\frac{2Z_{bot}}{Z_{s}+Z_{bot}}\right)$$

(21) ∴   p˜top′(t+dv)≠ p˜s′(t+xv)

It must be taken into account that in Equations (14)–(21), the mechanical losses of the sample (α(ω) and β(ω)) are not considered, which adds to the difference in the results of Figure 7a,b.

## 5. Acoustic Attenuation and Dispersion Coefficients Calculation

This method of known charge values at the dielectric bulk can be used for a direct measurement of the mechanical losses without the interference of the acoustic discontinuities at the external electrodes. This can be performed using two electrodes at the dielectric bulk which we can name s1 and s2 with a distance ∆x [m] between them, and by applying voltage values to generate equal charges at these electrodes, the generated pressure waves at these electrodes are equal:

(22) p˜s(t−xv)=p˜s(t−x+∆xv)

Because both pressure waves are generated at the dielectric bulk, the acoustic generation and transmission coefficients are equal for both electrodes ($a_{s})$:

(23) $$a_{s}=G_{s}T_{s-bot}$$

Following the same procedure as in Equations (2)–(6), the acoustic wave from each internal electrode arriving at the sensor is:

(24) $${\tilde{p}}_{s1}{}^{\prime }\left(t\right)=a_{s}F^{-1}\left[P\left(x,\omega \right)H\left(x,\omega \right)\right]$$

(25) $${\tilde{p}}_{s2}{}^{\prime }\left(t\right)=a_{s}F^{-1}\left[P\left(x+∆x,\omega \right)H\left(x+∆x,\omega \right)\right]$$

The measured ratio between the signal coming from each dielectric bulk electrode is the direct transfer function for the mechanical losses effect as derived in Equation (28):

(26) $$\frac{F[{\tilde{p}}_{s1}{}^{\prime }\left(t\right)]}{F\left[{\tilde{p}}_{s2}{}^{\prime }\left(t\right)\right]}=\frac{P\left(x,\omega \right)H\left(x,\omega \right)}{P\left(x+∆x,\omega \right)H\left(x+∆x,\omega \right)}$$

(27) $$\frac{F[{\tilde{p}}_{s1}{}^{\prime }\left(t\right)]}{F\left[{\tilde{p}}_{s2}{}^{\prime }\left(t\right)\right]}=\frac{e^{-\alpha \left(\omega \right)x}e^{-i\beta \left(\omega \right)x}}{e^{-\alpha \left(\omega \right)x+∆x}e^{-i\beta \left(\omega \right)x+∆x}}$$

(28) $$\frac{F[{\tilde{p}}_{s1}{}^{\prime }\left(t\right)]}{F\left[{\tilde{p}}_{s2}{}^{\prime }\left(t\right)\right]}=e^{-\alpha \left(\omega \right)∆x}e^{-i\beta \left(\omega \right)∆x}$$

Solving for α and β, gives us a direct value for the attenuation and dispersion values without the interference of the acoustic discontinuities at the external electrodes. The transfer function for attenuation and dispersions factors of Equations (26)–(28) was presented in [22,28]; By applying the proposed calibration sample, the transfer function can be calculated using the measuring external electrode and the internal electrode, avoiding the acoustic discontinuity at the external top electrode [19].

## 6. Equipment Characterization

The use of the calibrated samples presented in this paper can be used to compare and analyze the accuracy of space charge measuring systems (including equipment setup and post-processing analysis).

The procedure consists in performing short time duration measurements at the proposed calibrated samples. The short duration of the measurements is to avoid significant accumulation of space charges and only measure the surface charges induced at the electrodes. The resultant values of space spaces after the post-processing can then be compared with the pre-calculated values of charge density at each electrode of the calibrated sample. The deviation of the comparison reflects the accuracy of the measurement system under test.

## 7. Conclusions

The use of multilayer samples with electrodes at the layers interface can represent a single layer dielectric with known charge values in a localized region. The sample can be used for reference measurements and calibration of measuring equipment because of its capability of having known charges at each electrode. Because of the small thickness of dielectric-dielectric interface electrode, it can be considered as an area distribution of charges instead of a volume and use it to verify the spatial resolution of the space charge measuring system.

In this paper, the PEA method was used as an experimental test, but the multilayer samples with an interface electrode are expected to work for any acoustic or thermal method, but further research might be required.

The construction of these samples requires special attention to keep each layer with a constant thickness and to avoid the effect of the small distortion at the interface as in Figure 6.

A sample with several layers can be used to quantify the acoustic attenuation by comparison of the internal electrodes, without the interference of the acoustic impedance mismatch at the external electrode interfaces. The proposed method could apply to multilayer samples with different dielectric materials, but further research is required.

## Figures and Tables

**Table 1:** Geometric properties of the dual layer sample.

Property	Top Layer	Bottom Layer
Diameter	41.5 mm	55.8 mm
Thickness	0.91 ± 0.06 mm	0.67 ± 0.06 mm
Gold plating diameter	37.4 mm	37.4 mm

**Table 2:** Geometric properties of the single layer sample.

Property	Single Layer
Diameter	55.8 mm
Thickness	1.86 ± 0.06 mm
Gold plating diameter	37.4 mm
